# Direction-Specific Changes in Trunk Muscle Synergies in Individuals With Extension-Related Low Back Pain

**DOI:** 10.7759/cureus.54649

**Published:** 2024-02-21

**Authors:** Hiroki Saito, Hikaru Yokoyama, Atsushi Sasaki, Kimitaka Nakazawa

**Affiliations:** 1 Department of Physical Therapy, Tokyo University of Technology, Tokyo, JPN; 2 Division of Advanced Health Science, Institute of Engineering, Tokyo University of Agriculture and Technology, Tokyo, JPN; 3 Department of Physical Medicine and Rehabilitation, The Miami Project to Cure Paralysis, University of Miami Miller School of Medicine, Miami, USA; 4 Department of Life Sciences, Graduate School of Arts and Sciences, University of Tokyo, Tokyo, JPN

**Keywords:** electromyography (emg), functional tests, motor control, low back pain, trunk muscle synergies

## Abstract

Background

Identifying altered trunk control is critical for treating extension-related low back pain (ERLBP), a common subgroup classified by clinical manifestations. The changed coordination of trunk muscles within this group during particular trunk tasks is still not clearly understood.

Objectives

The objective of this study is to investigate trunk muscle coordination during 11 trunk movement and stability tasks in individuals with ERLBP compared to non-low back pain (LBP) participants.

Methods

Thirteen individuals with ERLBP and non-LBP performed 11 trunk movement and stability tasks. We recorded the electromyographic activities of six back and abdominal muscles bilaterally. Trunk muscle coordination was assessed using the non-negative matrix factorization (NMF) method to identify trunk muscle synergies.

Results

The number of synergies in the ERLBP group during the cross-extension and backward bend tasks was significantly higher than in the non-LBP group (p<0.05). The cluster analysis identified the two trunk synergies for each task with strikingly similar muscle activation patterns between groups. In contrast, the ERLBP group exhibited additional trunk muscle synergies that were not identified in the non-LBP group. The number of synergies in the other tasks did not differ between groups (p>0.05).

Conclusion

Individuals with ERLBP presented directionally specific alterations in trunk muscle synergies that were considered as increased coactivations of multiple trunk muscles. These altered patterns may contribute to the excessive stabilization of and the high frequency of hyperextension in the spine associated with the development and persistence of ERLBP.

## Introduction

Extension-related low back pain (ERLBP) is a common subgroup classified based on clinical manifestations, wherein pain is associated with extension-related spinal movements. These movements include sports activities and prolonged standing or sitting postures that excessively stabilize the lumbar region into the lordosis position [1-7]. Consequently, this can increase the load on the posterior elements of the spine, including the pars interarticularis [8] and facet joints [9], potentially leading to a specific source of nociceptive driver.

Within biopsychosocial frameworks, motor control assessment has emerged as a crucial physical and behavioral approach [4,10]. It quantifies an individual's strategy in controlling their body to modify the loading on the spine and adjacent structures [4,10,11]. The prevailing view supports identifying subgroups with distinct underlying mechanisms of trunk control strategies in the presence of low back pain (LBP). Adopting such an approach could improve the efficacy of clinical trials by shifting from standardized methods to more tailored treatment strategies for a clinically heterogeneous group of LBP patients, focusing on specific motor control dysfunctions [4,10,12]. Exercise and motor control approaches often target muscle activities to optimize spinal movement [13]. Thus, assessing specific alterations in muscle activity patterns that need to be addressed in individuals with ERLBP is essential.

Optimal trunk control involves the activation of several back and abdominal muscles in a coordinated manner in response to a specific demand that requires spinal stability and the movement of the spine [14]. Given the redundancy of the musculoskeletal system, the central nervous system (CNS) utilizes a few sets of motor modules or muscle synergies formed by a group of synchronously activated muscles as a simplified neural control strategy from a large subspace [15,16]. The determination of the identity of muscle synergies has relied on the analysis of multiple electromyography (EMG) activities based on dimensionality reduction algorithms, such as non-negative matrix factorization (NMF) [17-19]. The analysis provides an appealing method for evaluating the nature of motor impairment in movement disorders such as spinal cord injuries (SCI) and stroke [20-24]. For example, a previous study showed that individuals with SCI used a decreased number of trunk muscle synergies, representing the co-contractions of trunk muscles [24].

The purpose of the study is to investigate the differences in trunk muscle synergies between healthy participants and individuals with ERLBP. For the analysis of trunk muscle synergies, the previous study developed a series of 11 trunk-related functional motor tasks, including forward bending, backward bending, and rotation, which require different directions of spinal movement and stability to assess trunk muscle synergies [25]. Trunk task selection has been frequently used in the clinical and research fields [1-6]. The analysis of trunk muscle synergy underlying such a variety of tasks would comprehensively profile the trunk coordination strategy in the clinical heterogeneity of the LBP population that responds differently to the various mechanical demands of motor tasks [25]. As ERLBP exhibits directional-specific pain and dysfunction [1-7], we hypothesized that altered trunk muscle synergy patterns in the ERLBP subgroup would be particularly identical during trunk motor tasks that involved spinal extension and stability, rather than those involving other directions of spinal movement.

## Materials and methods

Participants

In this cross-sectional study, 13 individuals with ERLBP participated (six males and seven females). Individuals with ERLBP were considered to have experienced nonspecific LBP for longer than three months [26] and symptoms provoked by movement and postures involving the extension of the lumbar spine without pain provoked by movement involving spinal flexion [2,7,27]. A physiotherapist confirmed the pain-related spinal extension by active extension test with or without overpressure [28]. We did not establish specific numerical threshold values for pain assessment. Instead, the evaluation was based on the presence or absence of pain as reported by the participants during the active extension test. Overpressure was applied only when the active extension test did not induce pain, attempting to elicit pain through the therapist's manual techniques. Overpressure involves the therapist passively extending the lumber spine further, beyond the point of active movement. The control group was selected for age and sex matching, comprising 13 healthy individuals (average age: 20.8 years; six males and seven females). This selection aimed to ensure demographic alignment with the ERLBP group, thus reducing potential confounding factors related to age and sex disparities. Individuals without low back pain (non-LBP) were included in this group if they had no significant history of low back pain affecting their functionality or necessitating treatment from a healthcare professional.

The participants suspected of having non-musculoskeletal reasons for LBP, such as circulatory and respiratory diseases, were excluded from our study. Additionally, we ruled out those with LBP who had back pain extending to their legs or an acute exacerbation of their LBP due to performing motor tasks. The study was conducted in accordance with the Declaration of Helsinki and approved by the local ethics committee of the University of Tokyo (approval number: 746). Informed consent was obtained from all the participants, and the rights of the subjects were protected. The participants were not involved in the design of the study or did not contribute in any other way to the study. The authors had access to information that could identify individual participants during and after data collection.

Questionnaires

The participants with ERLBP completed the Roland-Morris Questionnaire (RMQ) to assess LBP-related disability [29]. The individuals with ERLBP also completed the short form of the Orebro Musculoskeletal Pain Questionnaire (OMPQ), which is designed to predict chronic pain, disability, and the likelihood of extended sick leave [30,31], as well as the Tampa Scale for Kinesiophobia (TSK), which assesses fear avoidance behaviors and beliefs [32,33]. Lastly, the Pain Self-Efficacy Questionnaire (PSEQ) was used to assess their confidence in managing pain [34].

Experimental procedures

**Figure 1:**
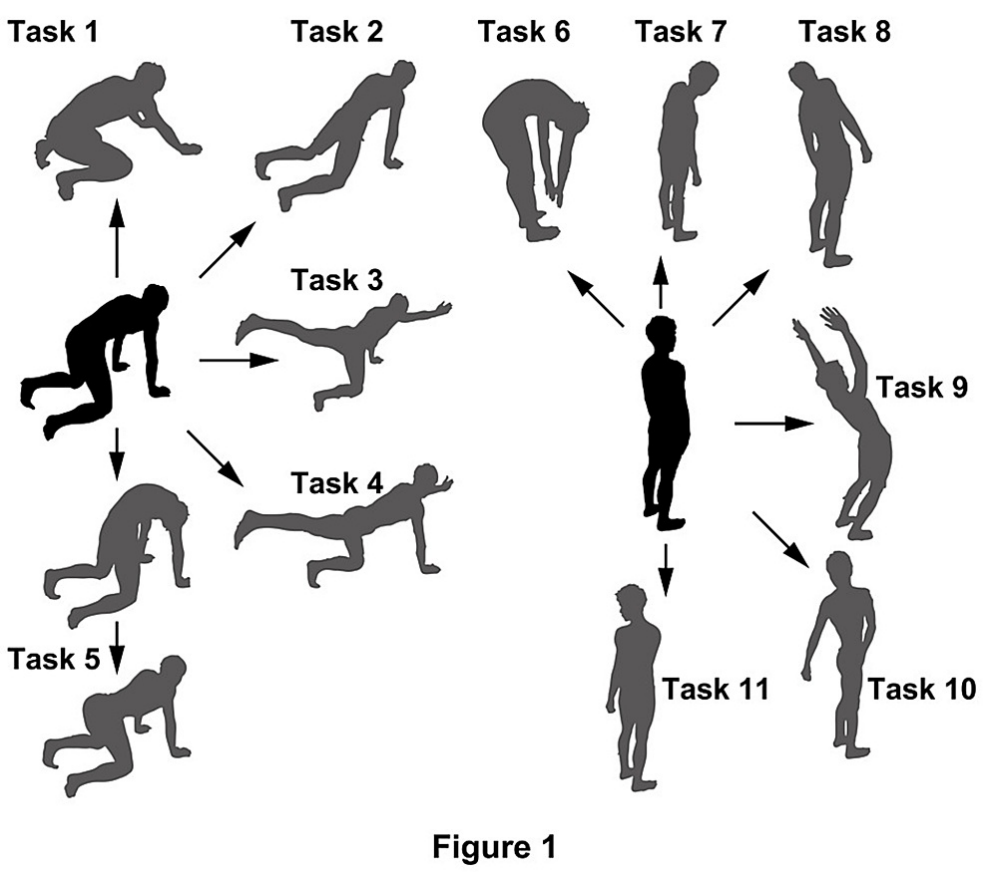
Eleven trunk motor tasks Stability tasks: rocking backward (task 1), rocking forward (task 2), cross-extension to the right (task 3), cross-extension to the left (task 4), and cat and dog (task 5). Movement tasks: forward bend (task 6), side bend to the right (task 7), side bend to the left (task 8), backward bend (task 9), rotation to the right (task 10), and rotation to the left (task 11). After the execution of the task, the participants were asked to return to the original starting positions (i.e., all four positions in tasks 1-5 and the normal standing position in tasks 6-11)

The participants were asked to freely perform 11 functional trunk tasks, as described in Figure 1. Each motor task is primarily employed in both research and clinical settings for the assessment and enhancement of motor control among individuals with LBP [6,35-37]. The tasks were performed eight times each, with the sequence of the tasks being assigned randomly.

Data collection

Surface EMG data were collected bilaterally from six back and abdominal muscles: erector spinae at Th1 (EST1), 5 cm lateral to the T1 spinous process [38]; erector spinae at Th9 (EST9), 5 cm lateral to the T9 spinous process [38]; erector spinae at L3 (ESL3), 3 cm lateral to the L3 spinous process [39]; latissimus dorsi (LD), located beside the T9 over the muscle belly [40]; rectus abdominis (RA), positioned 3 cm lateral to the navel [39]; and oblique externus (OE), 15 cm lateral to the navel [40]. We used a wireless EMG system (Trigno Wireless System, Delsys, Boston, MA) to measure EMG activity. Each electrode had an inter-electrode spacing of 10 mm. The EMG signals underwent band-pass filtering within the 20-450 Hz range, were amplified by a preamplifier with a gain factor of 300, and were recorded at a sampling rate of 1000 Hz. The initiation was marked by the examiner verbally saying "go" and manually pressing the electrical trigger a single time [41,42]. Once the participants had finished the tasks and settled into a rest position for about one second, the examiner signaled the conclusion of the movement by manually triggering the electrical device twice and issuing the verbal instruction "end" [41,42].

EMG processing

Raw EMG signals underwent a high-pass filter at 30 Hz to eliminate motion artifacts before the mean was removed from the data. Next, the signals were fully rectified and subjected to a low-pass filter at 10 Hz with a fourth-order Butterworth filter. Finally, to guarantee the equal contribution of the EMG data from each trial to the derived muscle synergies, the resulting smooth EMG envelopes were interpolated in time to produce 200 equidistant time points between the initiation and termination of each trial.

For each participant, we constructed individual EMG matrices tailored to each of the 11 trunk motor tasks, comprising data from 12 muscles over 1600 time points (calculated as eight repetitions multiplied by 200 samples per repetition) to isolate the muscle synergies related to each specific task. We normalized the EMG output of each muscle to its maximum amplitude observed across all tasks to maintain consistency. Subsequently, we standardized each muscle vector within the matrix to unit variance, ensuring that the contribution of each muscle activity was given equal importance.

The analysis of muscle synergy

Muscle synergies were derived by applying NMF to the single-task EMG matrix. NMF is a technique that has been characterized in prior studies as a method for linear decomposition, as per the following equation: M=W∙C+e (1), where M (an m×t matrix, where m represents the number of muscles and t the number of samples, indicating the spatiotemporal profiles of muscle activity) is the outcome of a linear combination of muscle synergies, W (an m×n matrix, where n is the number of muscle synergies) and C (an n×t matrix) represent the temporal patterns, and e is the matrix of residual error. We applied NMF to extract possible n values from 1 to 12 for each dataset. To determine the optimal count of muscle synergies, we calculated the variance accounted for (VAF) at each extraction [43,44]. We repeated each synergy extraction 50 times, computing the VAF in each case. Iterations yielding the highest VAF were retained [41,42,45,46]. We identified the optimal number of synergies using a VAF threshold of >0.9, a standard criterion in relevant literature [41,42,47-50].

We identified representative synergy vectors across the participants using hierarchical clustering analysis (Ward's method, Euclidean distance) of trunk muscle synergies for each task [46]. To determine the optimal number of clusters for each task in each group, if there was no significant difference in the number of muscle synergies between groups using the statistical analysis described below, the cluster analysis sorted the synergies into the rounded mean number of synergies of all the participants, including the non-LBP and ERLBP groups. Thus, in this case, the optimal number of clusters was the same for both groups. In contrast, if there was a significant difference in the number of synergies between the groups, the optimal number of clusters was the rounded mean number of synergies for each group. Subsequently, the muscle synergies in each cluster were averaged (synergy cluster centroid).

Sorting synergies based on similarity indices

Following the NMF and cluster analysis application for each group, the synergy cluster centroids would not have any pre-specified sequential order. Thus, functional sorting is necessary. First, we sorted the synergy cluster centroids in the non-LBP based on those in the ERLBP [51]. We calculated the scalar product for every possible pair of synergy cluster centroids between non-LBP and ERLBP. We chose the pair with the greatest similarity score and eliminated the corresponding synergy cluster centroids from the group. Following this, we selected the highest similarity score from the remaining groups and continued removing pairs in this manner until each synergy cluster centroid was paired with its most compatible match. As a result, synergies of the same order were found to be similar across both groups [50,52]. The similarity values (<0.8) between the best-matched synergy cluster centroids did not indicate that the two matched synergies between groups were similar [53].

Statistics

For each task, we compared the number of muscle synergies and VAF between the non-LBP and ERLBP groups. The values were compared using a permutation test with 1000 permutations [45,54], which is a nonparametric comparison because a normal distribution was not observed in the data (tested using the Shapiro-Wilk test). Corrections of p-values for multiple comparisons obtained from the test were performed using the false discovery rate (FDR) correction [55]. For all tests, a p-value of less than 0.05 was defined as the threshold for statistical significance. When there was significant difference in the number of muscle synergies between the groups for each task, effect size (ES) was calculated using Cohen's d [56].

Thirteen participants were enrolled without performing a power analysis beforehand. Consequently, a sensitivity analysis using G*Power (Heinrich Heine University Düsseldorf, Düsseldorf, Germany) was carried out, revealing that an effect size (ES) of 1.17 would be required to achieve 80% power with an alpha (α) level of 0.05.

## Results

Participants

Table 1 displays the baseline characteristics for the non-LBP and ERLBP groups. The current pain intensity for individuals with ERLBP was rated at 3.1±1.0, in contrast to the non-LBP group who reported no pain during the experiment. The average pain increase immediately following the experiment for the ERLBP group was 1.76±0.99.

**Table 1:** Demographic and clinical characteristics of the extension-related low back pain (ERLBP) and control (non-LBP) groups LBP, low back pain; NRS, numerical rating scale (0-10); RMQ, Roland-Morris Questionnaire (0-24); TSK, Tampa Scale for Kinesiophobia (17-68); OMPQ, Orebro Musculoskeletal Pain Questionnaire (0-100); PSEQ, Pain Self-Efficacy Questionnaire (0-60)

Characteristics	Non-LBP group (n=13)	ERLBP group (n=13)
Age (years)	21.3 (±0.6)	20.8 (±0.7)
Height (cm)	166.5 (±10.6)	165.3 (±9.3)
Weight (kg)	58.6 (±10.8)	58.6 (±8.2)
BMI	21.0 (±2.1)	21.2 (±1.8)
Sex (female/male)	6/7	6/7
Duration of pain (months)	-	44.4 (±25.0)
Average pain intensity during the day (NRS)	-	3.1 (±1.0)
Average increased pain immediately after the experiment	-	1.76 (±0.99)
RMQ	-	3.4 (±2.7)
TSK	-	40.0 (±10.1)
OMPQ	-	30.6 (±13.6)
PSEQ	-	32.7 (±7.9)

Trunk muscles synergies in the non-LBP and ERLBP groups

Table 2 presents the VAF values between the groups for each task. There were no significant differences in the VAF between the groups for all tasks when the optimal number of synergies was determined using our criteria (p>0.05).

**Table 2:** Variance accounted for (VAF) in each task for the extension-related low back pain (ERLBP) and control (non-LBP) groups LBP: low back pain

VAF values	ERLBP group (n=13)	Non-LBP group (n=13)	p
Task 1	0.92 (±0.01)	0.91 (±0.01)	0.55
Task 2	0.92 (±0.01)	0.91 (±0.01)	0.31
Task 3	0.91 (±0.01)	0.91 (±0.01)	0.30
Task 4	0.92 (±0.01)	0.91 (±0.01)	0.30
Task 5	0.92 (±0.01)	0.91 (±0.01)	0.96
Task 6	0.92 (±0.01)	0.91 (±0.01)	0.31
Task 7	0.91 (±0.01)	0.91 (±0.01)	0.73
Task 8	0.91 (±0.01)	0.91 (±0.01)	0.96
Task 9	0.92 (±0.01)	0.91 (±0.01)	0.20
Task 10	0.92 (±0.01)	0.91 (±0.01)	0.75
Task 11	0.92 (±0.01)	0.92 (±0.01)	0.96

**Figure 2:**
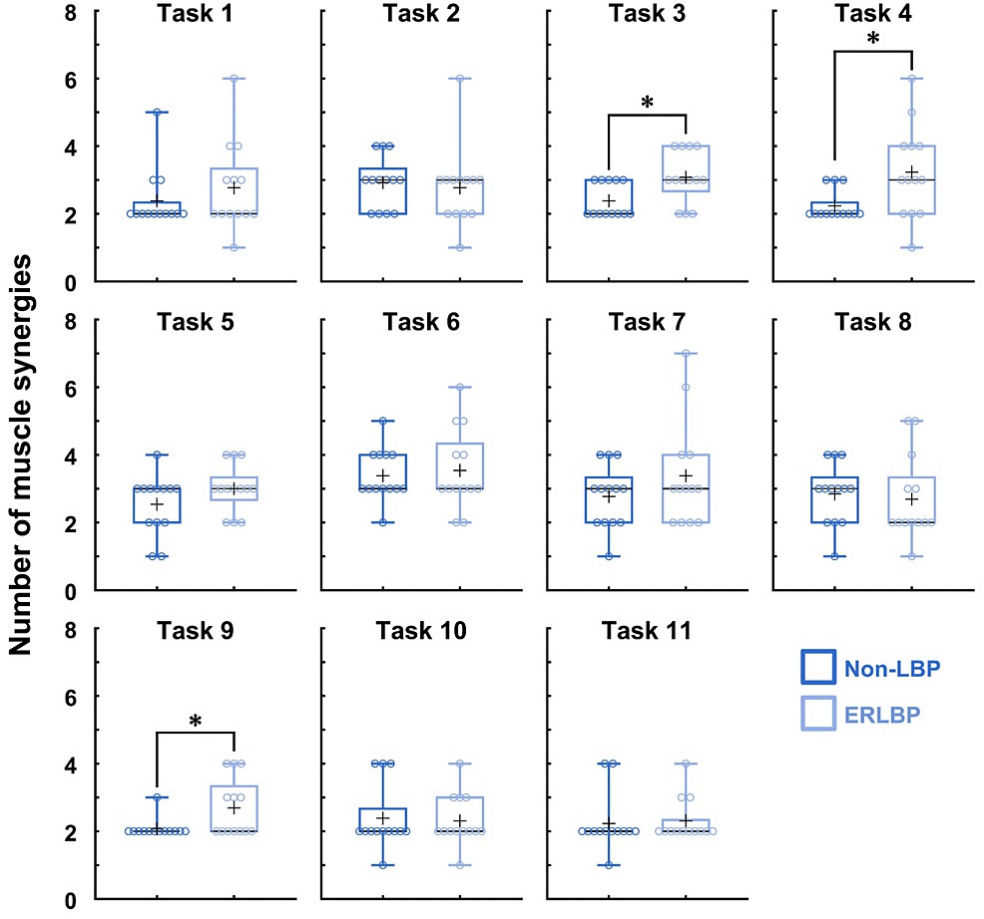
The number of trunk muscle synergies between the non-LBP and ERLBP groups for 11 trunk movement tasks The mean values are denoted by "+" and the median values by horizontal lines within the boxes. The 25th and 75th percentiles are represented by the box edges. For tasks 3, 4, and 9, a greater number of trunk muscle synergies, indicated by ✳︎, were observed in the ERLBP group ERLBP, extension-related low back pain; LBP, low back pain

Figure 2 compares the extracted number of synergies between the groups. Statistical analysis revealed that the number of synergies in the ESLBP group was significantly higher than in the non-LBP group for tasks 3, 4, and 9 (p<0.05; ES=1.07, 0.98, and 0.96, respectively). There were no significant differences in the number of synergies between the groups for the other tasks (p>0.05).

**Figure 3:**
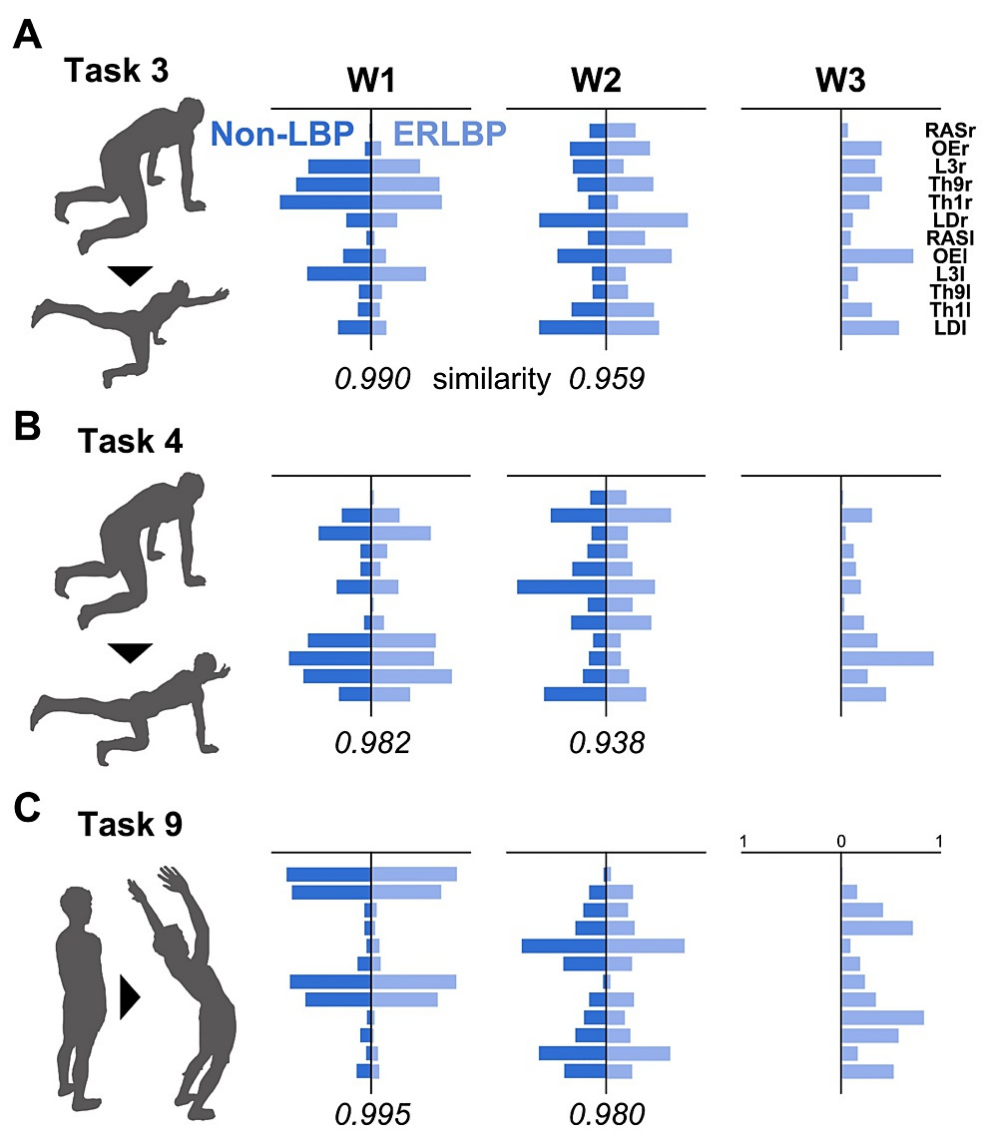
Comparisons of trunk muscle synergies between the non-LBP and ERLBP groups for (A) task 3, (B) task 4, and (C) task 9 Similarity calculated by scalar product is below the line between groups. Overall, two of the three trunk muscle synergies (W1 and W2) are strikingly similar (0.8>similarity). For each of the tasks, the ERLBP group shows the additional trunk synergy that is not identical in the non-LBP group (W3) ERLBP, extension-related low back pain; LBP, low back pain; RASr, rectus abdominis on the right side; OEr, oblique externus on the right side; L3r, erector spinae at L3 on the right side; Th9r, erector spinae at Th9 on the right side; Th1r, erector spinae at Th1 on the right side; LDr, latissimus dorsi on the right side; RASI, rectus abdominis on the left side; OEI, oblique externus on the left side; L3I, erector spinae at L3 on the left side; Th9l, erector spinae at Th9 on the left side; Th1l, erector spinae at Th1 on the left side; LDI, latissimus dorsi on the left side

Figure 3 shows the synergy cluster centroids in the non-LBP and ERLBP groups in tasks 3, 4, and 9, which showed significant group differences in the number of synergies.

**Figure 4:**
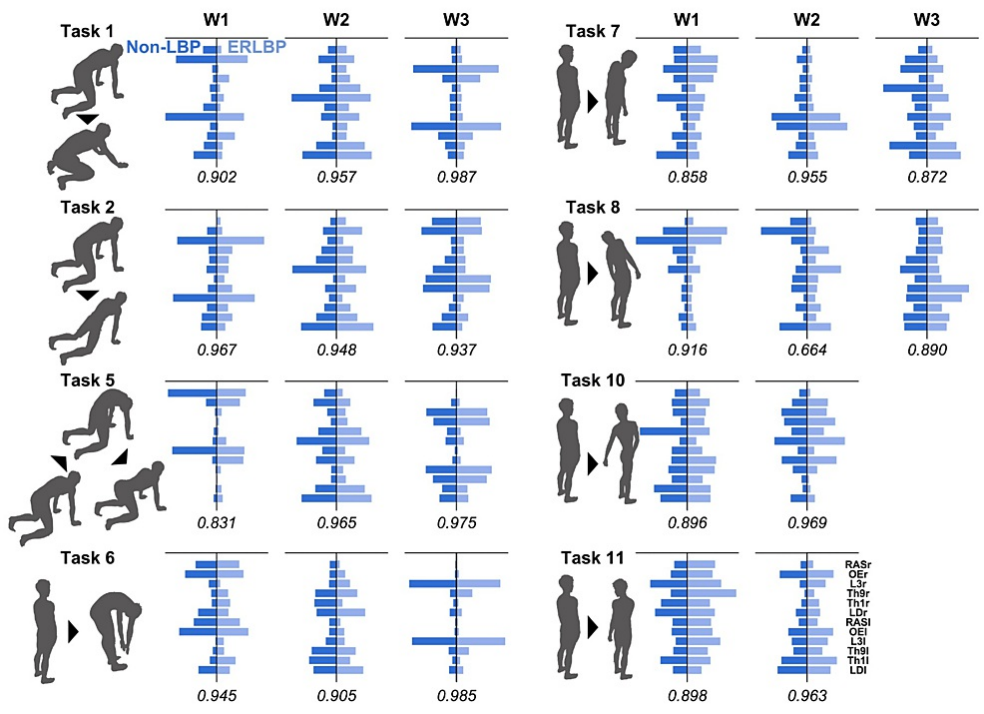
Comparisons of trunk muscle synergies between the non-LBP and ERLBP groups for trunk motor tasks (other than tasks 3, 4, and 9) Similarity calculated by scalar product is below the line between groups. For each task, trunk muscle synergies are similar (0.8>similarity) except W2 in task 8 (0.664 of similarity) ERLBP, extension-related low back pain; LBP, low back pain; RASr, rectus abdominis on the right side; OEr, oblique externus on the right side; L3r, erector spinae at L3 on the right side; Th9r, erector spinae at Th9 on the right side; Th1r, erector spinae at Th1 on the right side; LDr, latissimus dorsi on the right side; RASI, rectus abdominis on the left side; OEI, oblique externus on the left side; L3I, erector spinae at L3 on the left side; Th9l, erector spinae at Th9 on the left side; Th1l, erector spinae at Th1 on the left side; LDI, latissimus dorsi on the left side

The mean number of synergies in the non-LBP groups was 2.38 (±0.50), 2.23 (±0.43), and 2.07 (±0.27) for tasks 3, 4, and 9, respectively. Thus, the rounded mean number of synergies in the non-LBP group was 2, and the cluster analysis sorted the trunk muscle synergies in non-LBP into two groups for each task. In contrast, the mean synergies in the ERLBP groups were 3.07 (±0.75), 3.23 (±1.36), and 2.69 (±0.85) for tasks 3, 4, and 9, respectively. Thus, the rounded mean of synergies in the ERLBP group was 3, and the cluster analysis sorted the trunk muscle synergies in ERLBP into three groups for each of these tasks. The two trunk muscle synergies for each task were strikingly similar between groups (similarity values>0.9). Furthermore, the ERLBP group exhibited additional synergy for tasks 3, 4, and 9. Visual inspection revealed that these additional synergies presented the dominant unilateral erector spinae muscle and OE activation patterns in tasks 3 and 4 and the bilateral erector spinae muscle and OE patterns with higher activation of the lower erector spinae at Th9 and L3 in task 9. For other trunk motor tasks, almost all synergy cluster centroids showed high similarity between the groups (Figure 4).

## Discussion

We investigated altered trunk muscle synergies in the ERLBP group in a series of functional trunk motor tasks to comprehensively map the overall picture of the control strategy in the presence of LBP. The novelty of our work is to provide the first demonstration that (1) the number of trunk muscle synergies in the ERLBP group during cross-extension tasks (i.e., tasks 3 and 4) and backward bend task (task 9) is significantly higher than those in the non-LBP group and (2) the ERLBP group exhibited altered trunk muscle synergies in tasks 3, 4, and 9 while synergies in other tasks were strikingly similar between groups. Thus, the results support the original hypothesis that the ERLBP group showed altered trunk synergy patterns in tasks that included spinal extension and stability. We consider these additional synergies in the ERLBP group as the excessive activation of trunk muscles associated with the development or persistence of LBP.

ERLBP is defined as the group of individuals who have pain provoked by activities related to spinal extension movement and the position of lumbar lordosis [1-7]. The present study revealed specific trunk synergies (W3 in Figure 3) as the co-contraction of the different levels of the back and abdominal muscles for the trunk motor tasks that involved spinal stability (tasks 3 and 4) and the extension task (task 9). The current results confirm previous studies showing that individuals with ERLBP were characterized by the increased activation of the superficial back muscles, described as the absence of the flexion relaxation of the back muscles [57-59], and may contribute to protective behavior that presents less and slower spinal movement [60] and decreased spinal movement variability [6,12]. In the short term, this strategy can be an adaptive strategy to avoid nociceptive excitation, tissue injuries, the perception of pain, or the anticipation of such threats to prevent further injuries [12]. However, the synergies with the increase in the co-contraction of multiple trunk muscles lead to adverse mechanical consequences: the excessive stabilization of the spine and the increased frequency of hyperextension, which causes the accumulation of tissue stress in the lumbar region, driving the ongoing peripheral nociceptive inputs [57-59,61,62].

Furthermore, we also showed different muscle synergy patterns in ERLBP between tasks 3, 4, and 9, which had various biomechanical features. Thus, it is presumed that the CNS utilizes various adaptations of control strategies in the presence of LBP to respond to the specific mechanical demands of a task (Figure 2). For example, when the task required the stabilization of the spine with asymmetric upper and lower limb movements (i.e., tasks 3 and 4), the individuals with ERLBP exhibited excessive unilateral trunk patterns on the side of the lifting arm and balance on the knee (e.g., the specific synergy of the right trunk muscle patterns when lifting the right arm and balancing on the right knee in task 3). Likewise, when moving the trunk backward (bilateral spinal movement) in task 9, they showed excessive bilateral trunk muscle patterns. The findings of unilateral and bilateral excessive trunk muscle activation in different tasks may explain the mechanisms of bilateral and unilateral LBP symptoms depending on activities involving symmetric and asymmetric spinal movements and postures in clinical practice.

Based on the muscle synergy analysis, identifying the altered structure of muscle coordination patterns in the ERLBP group provides a basis for treatment. Specifically, treatment is directed at reducing the number of trunk synergies (i.e., reducing excessive trunk muscle activation patterns). Notably, we found that excessive trunk muscle patterns in the ERLBP group involved painful sites at lumbar levels and nonpainful sites at the thoracic level and in the abdominal region. Potential interventions include neuromuscular exercises with various movements and postures involving different trunk muscles [7,63-65]. For example, interventions that focus on improving trunk coordination by relaxing the trunk muscles to dissociate lumbopelvic movement from thoracic movement with proper breathing improve the flexion relaxation ratio of the trunk muscles [7] and LBP symptoms [7,66]. However, despite the widespread use of motor control approaches, clinical trials with such interventions rarely utilized muscle synergy analysis to objectively evaluate coordination patterns in the LBP population. Thus, future studies should incorporate an analysis of muscle synergies to provide rational and precise targets for specific LBP subgroups. Another aspect of the novelty of the present study is that it provides the possible ability to capture motor control deficits in the different subgroups of clinical heterogeneity in LBP that show different movement profiles by applying a series of trunk motor tasks. In this study, the ERLBP group showed only altered synergy patterns during stability and spinal extension tasks. In contrast, the flexion-related LBP subgroup could show altered synergy patterns in tasks with spine flexion, such as backward rocking (task 1) and forward bending (task 6) [1-7].

Our study had some limitations that may impact the interpretation and generalizability of our findings. Firstly, the relatively small sample size limit may affect the extrapolation of our results to other patient populations. Secondly, our analysis did not evaluate the behavior of the diaphragm muscle nor the deep trunk muscles such as the transverse abdominal muscle and internal oblique, all of which play a fundamental role in trunk stability and control by contributing to intra-abdominal pressure regulation [4,13,14]. The omission of these critical components means that we may have overlooked essential elements of trunk stability mechanisms, potentially influencing our understanding of the control strategies employed by individuals with ERLBP.

## Conclusions

This study showed more trunk muscle synergies in individuals with ERLBP. The excessive activation patterns of multiple back and abdominal muscles in the ERLBP group may lead to an increased frequency of the hyperextended position of the lumbar regions and thus contribute to the development of ERLBP. Neurophysiological indices with computational methodologies that reveal the structure of synergy organization could provide a powerful suite of motor control assessments and facilitate the development of novel interventions in individuals with LBP.
